# Molecular Cytogenetic Characterization of a Non-Robertsonian Dicentric Chromosome 14;19 Identified in a Girl with Short Stature and Amenorrhea

**DOI:** 10.1155/2012/212065

**Published:** 2012-11-05

**Authors:** Usha R. Dutta, Vijaya Kumar Pidugu, Ashwin Dalal

**Affiliations:** Diagnostics Division, Center for DNA Fingerprinting and Diagnostics, Tuljaguda Complex, 4-1-714, Andhra-Pradesh Hyderabad 500 001, Andhra-Pradesh, India

## Abstract

We report a 16-year-old girl who presented with short stature and amenorrhea. Initially the cytogenetic analysis showed the presence of a mosaic non-Robertsonian dicentric chromosome involving chromosomes 14 and 19. Subsequent molecular cytogenetic analysis by fluorescence *in situ* hybridization (FISH) using whole chromosome paints, centromeric probes, as well as gene specific probes confirmed the dicentric nature of the derivative chromosome and indicated that the rearrangement involved the short arms of both of these chromosomes. Furthermore, we also determined that the chromosome 19p13.3 breakpoint occurred within the terminal 1 Mb region. This is the first report of a mosaic non-Robertsonian dicentric chromosome involving chromosomes 14 and 19 with the karyotype determined as 45,XX,dic(14;19)(p11.2;p13.3)[35]/46,XX[15], and we suggest that the chromosome rearrangement could be the cause of clinical phenotype.

## 1. Introduction

Chromosomal abnormalities associated with disease phenotypes have often led to the identification of novel genes. Here we report on a girl with short stature and primary amenorrhea with a dicentric chromosome. Short stature refers to a height of a person which is below expected while, the term amenorrhea means absence of menstruation during puberty or later in life. The causes of amenorrhea include pregnancy, absence of uterus, vagina, hormonal imbalance, excess of male testosterone, endometritis, improper functioning of ovaries [1, 2], Mullerian agenesis [3], polycystic ovaries, gonadal dysgenesis, and also due to X/autosomal translocations [4]. Apparently the causes of both of these phenotypes are heterogeneous and may include either environmental or genetic factors. The genetic factors may be due to gene defects or due to chromosomal abnormalities involving both autosomes and sex chromosomes. There are several chromosomal abnormalities reported with short stature and amenorrhea independently but only one report describes the incidence of chromosomal abnormalities in females with both short stature and amenorrhea as 37.93% [5]. 

The structural chromosomal abnormalities like deletions, duplications, translocations, and so forth associated with various diseases are common but dicentric chromosomes in humans are rare [6] with only exception of Robertsonian translocations. The heterodicentric chromosomes involve centromeres derived from nonhomologous chromosomes [7]. These heterodicentric chromosomes have two alpha satellite sequences on the same chromosome which leads to a high risk of attachment of the same chromatid to the mitotic spindle from opposite poles and also in the formation of Anaphase Bridge during cell division. But in humans, dicentrics occur naturally in a substantial portion of the population and usually segregate successfully in mitosis and meiosis [8]. Their stability has been attributed to inactivation of one of the two centromeres, creating a functionally monocentric chromosome that can segregate normally during cell division or when the centromeres are very close to each other and form only one heterochromatic block [9]. 

To date there are approximately around 30 cases of non-Robertsonian dicentric chromosomes reported in a review by Lemyre et al. [9], including a few cases involving chromosomes 14 and 19 with other autosomes, for example, a case of primary amenorrhea showing a dic(12; 14) [10], dic(19; 20) [11], a 32 weeks growth retarded male fetus with a phenotype of trisomy 18 showed a mosaic karyotype of +18,dic(14; 18)/dic(14; 18). The mother carried the dicentric chromosome [9] and a 18q-syndrome resulting from dic(14; 18) [12]. But there are no reports of non-Robertsonian dicentric chromosome formation involving both chromosomes 14 and 19. In this paper, we describe a girl with short stature and amenorrhea and a non-Robertsonian dicentric chromosome 14; 19. 

## 2. Materials and Methods

### 2.1. Clinical Report

A 16-year-old girl was referred to our centre with short stature and amenorrhea. The detailed family history and written consent were taken from the patient and her family. She is the first child of the four children born at term to a nonconsanguineous couple. Birth history was uneventful; her birth weight was 2.5 kg. Clinical examination revealed absence of breasts, delayed secondary sexual character development, and reduced LH (Luteinizing hormone) levels. Her height was 131 centimeters (much below the 5th percentile) and no growth was observed in the last 4 years. Her father was 167 cms tall and her mother was 152 cms. Her 15-year-old sister was 153 cm and her two brothers aged 12 and 10 years were 149 and 140 cms. 

### 2.2. Cytogenetic Analyses

Chromosome analyses were performed on peripheral blood lymphocytes from the patient and her parents by using standard methods. Fifty metaphases were analyzed by GTG-banding using trypsin and Giemsa and also silver staining of the nuclear organizing regions (NORs) was done. 

### 2.3. Fluorescence *In Situ* Hybridization

FISH was performed with commercially available WCP probes for chromosomes 14 and 19 (Applied Spectral Imaging), centromeric probes SE (14/22) and SE (19/5/1), gene specific probe *ERCC1* (19q13), and *ZNF443* (19p13) (Kreatech, Netherlands) according to the manufacturer's instructions on metaphase spreads of the patient by using standard procedures.

Additional analysis was performed by using the BAC clone RP11-878J15 identified from 19p13.3 region according to the current human NCBI Reference Sequence [13] utilizing the Ensembl and UCSC Genome browser [14]. This clone was kindly provided by Dr. Vera Kalscheuer from the Max Planck Institute for Molecular Genetics, Berlin, Germany. BAC DNA was isolated using the NucleoBond Plasmid Midi kit (Macherey-Nagel, Dueren, Germany) and was labeled with biotin-16-dUTP (Roche Diagnostics, Mannheim, Germany) by nick translation. FISH analysis was performed on the patient metaphase chromosomes as described by standard protocols [15].

### 2.4. Polymerase Chain Reaction

DNA was extracted from peripheral blood of the patient by using a standard protocol. All exons and exon-intron boundaries of the *KISS1R*-derived peptide receptor *GPR54* gene were amplified by PCR using 20–100 ng of genomic DNA. The following primers were used. Exon 1: forward, GGGCGGCCGGGAGGAGGA; reverse, CCGGGACGGCAGCAGGTG. Exon 2: forward, GCCCAGCGCCCGCGCATC; reverse, GTCCCCAAGTGCGCCCTCTC. Exon 3: forward, CAGGCTCCCAACCGCGCAG; reverse, CGTGTCCGCCTTCTCCCGTG. Exon 4: forward, CTTCATCCTGGCTTGTGGCAC; reverse, CTTGCTGTCCTCCCACCCAC. Exon 5: forward, GCCTTTCGTCTAACCACCTTC; reverse, GGAGCCGCTCGGA-TTCCCAC. PCR amplification was performed for 35 cycles with 5U *Taq* (Fermentas) in 1.5 mM MgCl_2_, 0.1 M of each primer and with 4 *μ*L of Q Solution (Qiagen). The annealing temperature was 60°C for exons 1, 3, 4, and 5 and 66°C for exon 2. The PCR products were directly sequenced using the BigDye dideoxy terminator cycle sequencing kits with the same primers described above and were run on a 3130 Genetic analyzer (Applied Biosystems).

## 3. Results

**Figure 1:**
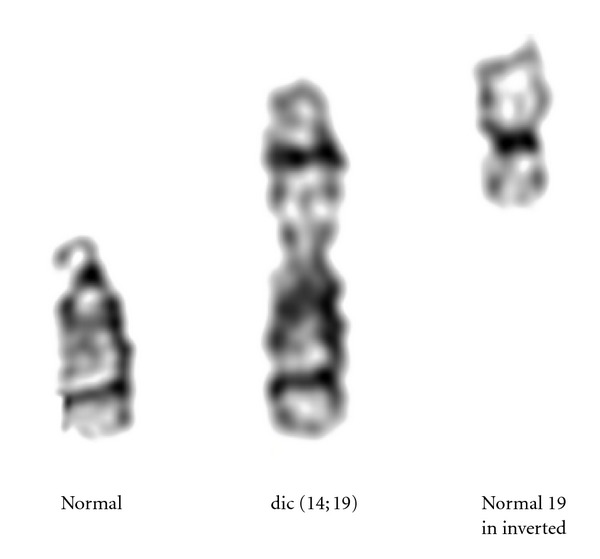
The partial karyogram showing the normal chromosome 14, dicentric 14 and 19, and normal chromosome 19.

**Figure 2:**
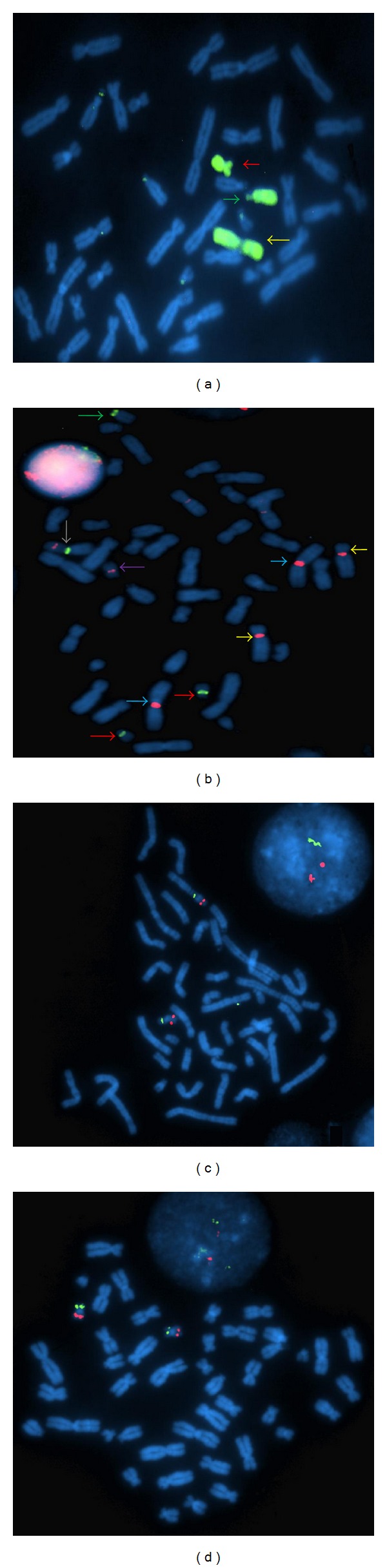
FISH on the lymphocyte metaphase spreads of the patient. (a) WCP FISH with 14 and 19 paints. Green arrow points normal 14, red arrow points the normal 19 and the yellow arrow points the derivative chromosome 14. (b) FISH with SE (14/22) and SE (19/5/1) shows green arrow on normal 14, violet on normal 19 and white arrow shows the dicentric 14. (c) FISH with *ZNF443* (19p13) *ERCC1* (19q13)/shows the green signals proximal to the dicentric chromosome and red signals towards the distal region. (d) *ERCC1* probe on normal metaphases shows both the signals on both the chromosome 19.

Cytogenetic analysis of the GTG banded chromosomes from the patient revealed a mosaic karyotype of 45,XX,dic(14;19)(p11.2;p13.3)[35]/46,XX[15]. (Figure 1). Chromosomes of both parents were normal. NOR staining showed the absence of satellites on the non-Robertsonian dicentric chromosome (data not shown). WCP FISH confirmed the involvement of chromosomes 19 and 14 in the rearrangement (Figure 2(a)). The dicentric nature of the derivative chromosome was confirmed by using centromeric probes (Figure 2(b)). As chromosome 19 is metacentric in origin, we took advantage of the FISH probes *ERCC1* and *ZNF443* which allowed us to determine the involvement of 19p or 19q arm in the rearrangement. FISH with this probe showed green signals on the proximal region and red signals on the distal region of the non-Robertsonian dicentric chromosome. From this result we concluded that the break occurred in 19p13.3 region (Figures 2(c) and 2(d)).

### 3.1. Assigning the Breakpoint on the Derivative Chromosome

**Figure 3:**
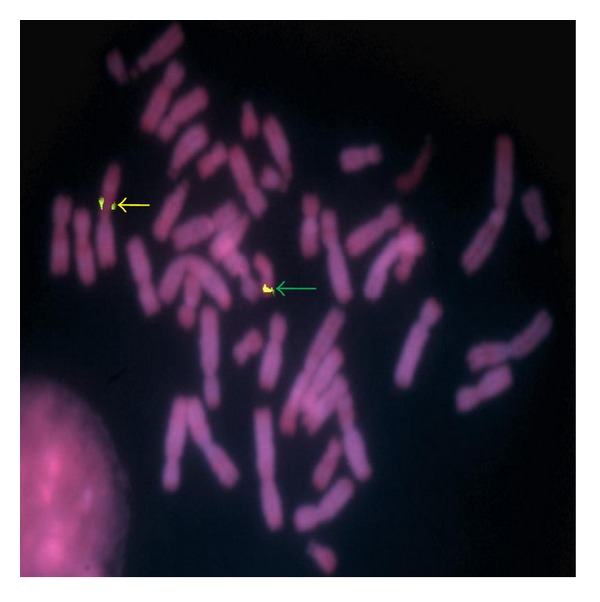
FISH with BAC clone RP11-878J15 on lymphocyte metaphase spreads of the patient. The signals in green (FITC) are shown by green arrow on normal 19 chromosome and the signals with yellow arrow points the derivative chromosome 14 on 19p13.3 region.

Once the breakpoint was confirmed on 19p region, we performed FISH with the BAC clone RP11-878J15 (959,520–1,144,508) (GRCh37/hg19) on 19p13.3 region. Signals were present both on normal chromosome 19 and on the non-Robertsonian dicentric chromosome (Figure 3). This result suggested that the breakpoint lies distal to this region.

**Figure 4:**
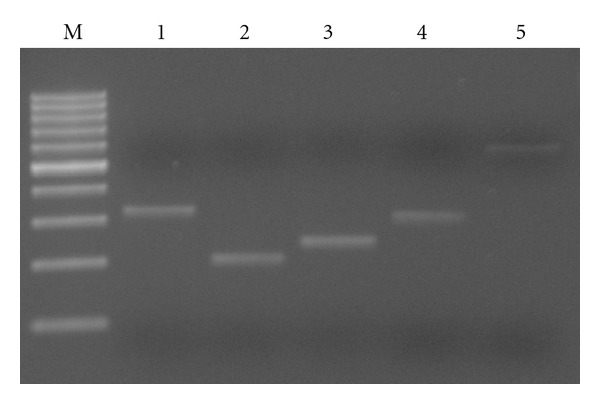
The gel showing the 5 exons of *KISS1R* gene.

Parallely, we had searched the databases for potential disease causing genes in the 19p13.3 region and identified *KISS1R* gene (917,342– 921,015) which appears to play a role in the onset of puberty. Hence we also analyzed for disease causing deletions or mutation in this gene by PCR with primer pairs flanking all the 5 exons and subsequent Sanger sequencing using the same primers (Figure 4). The sequence analysis did not reveal any pathogenic mutation in *KISS1R* gene and thereby excluded that sequence changes in this gene contributed to the patient's phenotype. These results suggest that the breakpoint lies in the approximate 1Mb telomeric to this region.

## 4. Discussion

Dicentric autosomes are not common in humans because they often result in partial trisomy and partial monosomy [6]. Except in Robertsonian translocations, autosomal constitutional dicentric chromosomes are rare and the majority of these involve an acrocentric chromosome with a short arm breakpoint fused to another chromosome in which there is a single breakpoint [16]. The dicentric chromosome formation is speculated to be due to meiotic recombination within a paracentric inversion loop, isochromatid break with U shaped rejoining, mitotic crossover, Robertsonian translocation and nonhomologous non-Robertsonian translocation.

The dicentric chromosomes are inherently unstable because of bridge formation and breakage during cell division. But the stability of dicentric chromosomes involving an acrocentric chromosome could be due to relatively small centromeric distance between the acrocentric chromosome and the autosome. Moreover, absence of a phenotype related to a deletion of the p arm of an acrocentric chromosome is also in favour of embryonic viability. The other reason for the stability could be due to inactivation of one of the two centromeres, creating a functionally monocentric chromosome that can segregate normally during cell division. The molecular mechanism of centromere inactivation is poorly understood, but studies suggest that genomic and epigenetic mechanisms can be involved. It was also observed that the stabilization of the centromere inactivation involves partial deletion of alpha satellite array of the inactive centromere [8].

We have studied a girl with short stature and amenorrhea and identified a non-Robertsonian dicentric chromosome 14 and 19. In majority of the cytogenetically recognizable heterodicentric autosomes, only one primary constriction is seen. The primary constriction corresponds to the activity of the centromere [17]. In our patient we had observed cytogenetically that centromere 19 lacked a constricted appearance where as centromere 14 showed a primary constriction on the metaphase chromosomes depicting that 14 is the active centromere. The inactive and active status of the centromere was also interpreted in a dic(13; 17) case by FISH [18]. Similarly, in our FISH experiments with the centromere probes we observed that the 19 centromere consistently showed a “double chromatid” signals whereas the 14 centromere showed single signal more like a monocentric chromosome. This suggests that the chromosome 19 centromere is suppressed and the centromere 14 is active. Also Sullivan et al. [19] showed in many Robertsonian translocation patients that chromosome 14 remained active most often, irrespective of the other acrocentrics involved. These results imply that some centromeres are “stronger” or less amenable to the centromere inactivation. Thus, we assume that our patient shows a pseudodicentric non-Robertsonian derivative chromosome. Hence, we speculate that the stability of the dicentric chromosome might have been achieved by inactivation of centromere 19. The mechanism would most likely be nonhomologous non-Robertsonian translocation.

Keeping in view the phenotypic features of the patient, we searched for potential candidate gene in 19p13.3 region. We confirmed the presence of *KISS1R* gene, and the protein encoded by this gene is a gelatin like G protein coupled receptor that binds metastin, a peptide encoded by the metastasis suppressor gene KISS1. The tissue distribution of the expressed gene suggests that it is involved in the regulation of endocrine function, and this is supported by the finding that this gene appears to play a role in the onset of puberty. Mutations in this gene have been associated with hypogonadotropic hypogonadism; De Roux et al. [20] showed the involvement of *KISS1R* in the regulation of gonadotropin secretion. This gene was present distal (917,342–921,015) to the BAC clone we had studied and within the 917 kb distance from the telomere. Our patient showed no deletions in this gene; hence, we assume that the breakpoint occurred distal to this region and could be within the terminal 917 kb region. It was also experimentally demonstrated that telomere disruption results in nonrandom formation of de novo dicentric chromosome involving acrocentric human chromosome [21]. Hence, the phenotype could be due to the disruption of sequences in the telomeric region or could be due to the position effect of some other gene located far from the breakpoint region. Further fine mapping and precise characterization of the breakpoint region may help in the identification of new genes involved in short stature and amenorrhea.

In summary the patient reported here increases the knowledge about non-Robertsonian dicentric chromosome with short stature and amenorrhea. We identified a non-Robertsonian dicentric chromosome and confirmed it and also excluded the involvement of *KISS1R* gene. It is always interesting to study cases as each case is a potential material for the elucidation of the genotype-phenotype correlation. To the best of our knowledge, this is the first patient with non-Robertsonian dicentric chromosome 14; 19 with short stature and amenorrhea.
